# Cell-Free Fetal DNA and Cell-Free Total DNA Levels in Spontaneous Abortion with Fetal Chromosomal Aneuploidy

**DOI:** 10.1371/journal.pone.0056787

**Published:** 2013-02-15

**Authors:** Ji Hyae Lim, Min Hyoung Kim, You Jung Han, Da Eun Lee, So Yeon Park, Jung Yeol Han, Moon Young Kim, Hyun Mee Ryu

**Affiliations:** 1 Laboratory of Medical Genetics, Medical Research Institute, Cheil General Hospital and Women's Healthcare Center, Seoul, Korea; 2 Department of Obstetrics and Gynecology, Cheil General Hospital and Women's Healthcare Center, KwanDong University College of Medicine, Seoul, Korea; University of Bonn, Institut of experimental hematology and transfusion medicine, Germany

## Abstract

**Background:**

Cell-free fetal DNA and cell-free total DNA in maternal circulation have been proposed as potential markers for noninvasive monitoring of the placental condition during the pregnancy. However, the correlation of and change in cell-free fetal DNA and cell-free total DNA in spontaneous abortion (SA) with fetal chromosomal aneuploidy have not yet been reported. Therefore, we investigated cell-free fetal DNA and cell-free total DNA levels in SA women with fetal chromosomal aneuploidy.

**Methodology/Principal Findings:**

A nested case-control study was conducted with maternal plasma collected from 268 women in their first trimester of pregnancy. Subjects included 41 SA with normal fetal karyotype, 26 SA with fetal chromosomal aneuploidy, and 201 normal controls. The unmethylated *PDE9A* gene was used to measure the maternal plasma levels of cell-free fetal DNA. The *GAPDH* gene was used to measure the maternal plasma levels of cell-free total DNA. The diagnostic accuracy was measured using receiver-operating characteristic (ROC) curves. Levels of cell-free fetal DNA and cell-free total DNA were significantly higher in both SA women with normal fetal karyotype and SA women with fetal chromosomal aneuploidy in comparison with the normal controls (*P*<0.001 in both). The correlation between cell-free fetal DNA and cell-free total DNA levels was stronger in the normal controls (r = 0.843, *P*<0.001) than in SA women with normal karyotype (r = 0.465, *P* = 0.002) and SA women with fetal chromosomal aneuploidy (r = 0.412, *P* = 0.037). The area under the ROC curve for cell-free fetal DNA and cell-free total DNA was 0.898 (95% CI, 0.852–0.945) and 0.939 (95% CI, 0.903–0.975), respectively.

**Conclusions:**

Significantly high levels of cell-free fetal DNA and cell-free total DNA were found in SA women with fetal chromosomal aneuploidy. Our findings suggest that cell-free fetal DNA and cell-free total DNA may be useful biomarkers for the prediction of SA with fetal chromosomal aneuploidy, regardless of fetal gender.

## Introduction

Spontaneous abortion (SA) is a common complication of pregnancy. It is defined as the loss of a fetus prior to 20 weeks of gestation. Approximately 15∼20% of clinically recognized pregnancies are spontaneously aborted, most of them during the first trimester [1]. The major cause of SA is fetal chromosomal abnormalities, contributing to about 50∼60% of the cases [2]. The most prevalent abnormality of SA is chromosomal aneuploidy [2]. Other etiologic factors of SA are anatomic anomalies, endocrine or hormonal problems, coagulation protein defects, and nutritional and environmental factors. At present, maternal serum b-hCG levels and sonographic parameters such as fetal heart rate (FHR) and crown-rump length (CRL), either used alone or in combination, are used for prognosis and diagnosis of SA [3]–[6]. However, up to 20% of pregnancies miscarry after the visualization of FHR by ultrasonography [7], and measurement of CRL can have a large deviation with merely a few millimeters. Therefore, the use of these parameters for prenatal diagnosis and treatment is controversial.

Cell-free fetal DNA and cell-free total DNA in maternal blood have been proposed as potential markers for noninvasive monitoring of the pregnancy condition. In previous studies, it was reported that cell-free fetal DNA and cell-free total DNA increased in association with various pregnancy-associated complications including pre-eclampsia, intrauterine growth restriction, preterm labor, placenta accreta, invasive placenta, fetomaternal hemorrhage and intrauterine fetal death [8]–[14]. Specifically, the maternal plasma levels of cell-free fetal DNA and cell-free total DNA were increased by about 4∼5 fold in pregnant women with SA compared with normal pregnant women [15], [16]. Therefore, cell-free fetal DNA and cell-free total DNA in maternal circulation have been suggested as valuable indicators for noninvasive diagnosis and prognosis of SA. However, in prior studies, prediction of SA has been limited to pregnant women with a male fetus due to the use of markers specific to the Y chromosome, such as *DYS14* and *SRY*, in the measurement of cell-free fetal DNA [15], [16]. Moreover, the correlation of and change in cell-free fetal DNA and cell-free total DNA in SA with fetal chromosomal aneuploidy have not yet been reported.

Recently, genomic regions that are differentially methylated between the placenta and the maternal blood cells have been considered as fetal-specific epigenetic makers to detect cell-free fetal DNA in maternal plasma. Because the cell-free fetal DNA in the maternal plasma is derived from the fetal placental cells such as trophoblast and the cell-free maternal DNA in the maternal plasma is predominantly derived from the maternal hematopoietic cells [17]–[19]. This epigenetic strategy has led to the identification of DNA sequences which are methylated differently in maternal blood and placenta. Chim et al. and Lim et al. reported that the region of human *PDE9A* gene, located on chromosome 21q22.3, is completely methylated in the maternal blood (*M-PED9A*) and unmethylated in fetal (placental) tissues (*U-PED9A*) [20]–[22]. In previous studies, CpG sequences of *U-PDE9A* were modified by bisulfite conversion and selectively amplified by quantitative methylation-specific PCR (qMSP) [21], [22]. Therefore, level of cell-free fetal DNA in maternal plasma could be measured without contamination of cell-free maternal DNA using fetal-specific epigenetic marker such as *U-PDE9A*. Moreover, it was useful to detect an increase of cell-free fetal DNA derived on chromosome 21 by fetal trisomy 21 independent of fetal gender [21]. Therefore, it is suggested that the *U-PDE9A* may be a useful marker in noninvasive prenatal diagnosis of fetal trisomy 21 [20], [21]. This might be possible because methylation pattern at the *U-PDE9A* region was not changed according to aneuploidy status and gestational period.

In the present study, the cell-fetal fetal DNA levels in maternal plasma is detected by qMSP of the *U-PDE9A*, while the cell-free total DNA is measured by quantitative real-time PCR of the *GAPDH* gene. We investigated the correlation between cell-free fetal DNA and cell-free total DNA in SA with fetal chromosomal aneuploidy and determined whether cell-free fetal DNA and cell-free total DNA levels could be used to predict SA with fetal chromosomal aneuploidy.

## Materials and Methods

### Ethics statement

This study was conducted according to the principles expressed in the Declaration of Helsinki. Appropriate institutional review board approval was obtained from the Ethics Committee at Cheil General Hospital for this study (#CGH-IRB-2011-85). Written informed consent was obtained from each participant before blood draws for the collection of samples and subsequent analysis.

### Sample collection and processing

We performed a nested case-control study of women who enrolled in the Cheil General Hospital Noninvasive Prenatal Diagnosis Study (CNPD). Participants in the CNPD were recruited from October 2008 for noninvasive prenatal diagnosis of rare and incurable fetal diseases. Participants were women who received prenatal care at Cheil General Hospital. Maternal blood samples were obtained from all participants at or before 12 weeks of gestation. Before maternal blood sampling, ultrasonography was recommended to establish the viability of each singleton pregnancy and to confirm the gestational age calculated from the time of last menstruation. Maternal, fetal, and infant records were collected prospectively and maintained in an electronic database.

The case group consisted of 67 women whose pregnancies spontaneously terminated prior to 20 weeks of gestation. For analysis of the differences in fetal chromosomal aneuploidy of SA, women in the case group were divided into two subgroups: SA with fetal chromosomal aneuploidy (n = 26) and SA with fetal normal karyotype (n = 41). Each case was paired with three controls that were matched according to gestational week at blood sampling. The control group consisted of 201 healthy pregnant women who delivered a healthy neonate at term (37 weeks of gestation or more) without miscarriage, fetal chromosomal abnormality, prematurity, stillbirth, eclampsia, or other pregnancy complications.

Ten milliliters of peripheral blood was obtained using ethylenediaminetetraacetic acid (EDTA) as an anti-coagulant. Immediately after blood sampling, plasma was separated from whole blood by centrifugation at 2,500 g for 10 min. Recovered plasma was then centrifuged for an additional 10 min at 16,000 g to minimize any additional release of maternal DNA. Circulating fetal DNA from 1 mL of maternal plasma was extracted using the QIAamp DSP Virus Kit (Qiagen, Hilden, Germany) according to the manufacturer’s instructions. The DNA was eluted into 30 µL sterile, DNase-free water. The samples were coded for subsequent blinded analysis.

### Cytogenetic Analysis for detection of aneuploidy

Chromosomal analyses of abortus samples were carried out using standard protocols [23], [24]. Cells from abortuses were cultured in the AmnioMAX-C100 culture medium (Invitrogen, CA, USA). Metaphase chromosomes were stained using the GTG-banding method, and 20 metaphases per sample were analyzed.

### Detection of cell-free fetal DNA in maternal plasma by qMSP

The *PDE9A* gene was found to be completely methylated in maternal blood cells and unmethylated in placentas obtained from both the first and third trimesters [20]. We previous confirmed this epigenetic characteristic of *PDE9A* gene by bisulfite sequencing [21], [22].The qMSP assay for *U-PDE9A* was performed to detect cell-free fetal DNA as described previously [20]–[22].

In brief, DNA extracted from 1 mL of maternal plasma was bisulfite-converted using the EZ DNA methylation kit and eluted with 25 µL of DNase-free water. The eluted DNA was used as the template for each PCR reaction. The qMSP assay for *U-PDE9A* was performed with a primer set and a dual-labeled fluorescent hydrolysis probe. Sequences of primers and probe were as follows; forward primer: 5′- GGT TTG TTT TGG TGA GTG TGT GTC GT-3′, reverse primer: 5′- CCC AAC CAT CCC AAA AAA GCA-3′, and probe: 5′-(FAM)-TTT GTT TGG TGA TGT TAT GTG GTT T-(MGBNFQ)-3′. The PCR reaction solution contained 12.5 µL iQ Supermix (Bio-Rad Laboratories, Hercules, USA), 200 nM hydrolysis probe (Applied Biosystems, Foster, USA), 400 nM primers, and 5 µL converted DNA per 25 µL total reaction volume. The thermal profile for the qMSP assay consisted of an initial denaturation step of 95°C for 10 min followed by 50 cycles of 95°C for 15 sec, 60°C for 30 sec, and 72°C for 30 sec. Calibration curves for the assay were prepared by serial dilutions of single-stranded synthetic DNA oligonucleotide calibrators specific to *U-PDE9A*. The calibrator sequence was as follows; 5′-GGT TTG TTT TGG TGA GTG TGT GTT GTG GGT TTT GTT TGG TGA TGT TAT GTG GTT TTT TTG TTT TTT TGG GAT GGT TGG G-3′.

### Detection of cell-free total DNA in maternal plasma by quantitative real-time PCR

The *GAPDH* gene was used as the molecular marker for the quantification of cell-free total DNA extracted from maternal plasma such as previous studies [25], [26]. The PCR reactions were set up in a volume of 25 µL, using 12.5 µL of iQ Supermix (Bio-Rad Laboratories, Hercules, USA) and 5 µL of the extracted plasma DNA. Primers and probes were used at final levels of 300 nM and 100 nM for *GAPDH*, respectively. The sequences of the primers and a dual-labeled fluorescent hydrolysis probe were as follows: forward primer: 5′-CCC CAC ACA CAT GCA CTT ACC-3′, reverse primer: 5′-CCT AGT CCC AGG GCT TTG ATT-3′, and probe: 5′-(HEX)-AAA GAG CTA GGA AGG ACA GGC AAC TTG GC-(TAMRA)-3′. Initial denaturation cycling conditions were 95°C for 10 minutes, followed by 50 cycles of 15 seconds at 95°C and 1 minute at 60°C. The level of cell-free total DNA that was present in the plasma sample was determined by comparison with a standard dilution curve using a known level of a commercial genomic DNA (Promega, Madison, USA).

### Limit of detection for quantitative PCR

The mean cell-free fetal DNA concentration in maternal plasma was previously estimated to be 25.4 copies/mL (range: 3.3–69.4) in early pregnancy [27]. In qMSP of the *U-PDE9A* gene, 1 mL of maternal plasma is extracted, eluted in 25 µL, and then 5 µL is used for each PCR. Thus, each PCR has fetal DNA of > 5 copies. In quantitative real-time PCR, 1 mL of maternal plasma is extracted, eluted in 30 µL, and then 5 µL is used for each PCR. Thus, each PCR has fetal DNA of > 4 copies. Therefore, a PCR is sensitive enough because ≥ 3 copies of the target are required at a real-time PCR, according to the minimum information for publication of quantitative real-time PCR experiments guidelines [28]. Minimum value to consider the presence of circulating fetal and total DNA was 20 copies/mL for *U-PDE9A* and 1,000 copies/mL for *GAPDH*, as our previous method [22].

### Statistical Analysis

Data are expressed as a mean ± standard deviation (S.D.) or number (%). The clinical data and levels of cell-free fetal DNA and cell-free total DNA in the study groups were compared using robust one-way analysis of variance (ANOVA), the post hoc Tamhane’s T2 test for multiple comparisons, and the χ^2^-test. The accuracy of predicting SA during the first trimester was analyzed using the receiver operating characteristics (ROC) curve. Overall accuracy was estimated using the area under the ROC curve (AUC) with a 95% confidence interval. The optimal cutoff for each factor was set at 80% sensitivity. The specificity, positive predictive value (PPV), and negative predictive value (NPV) were calculated to determine the diagnostic efficiency using the EpiMax Table Calculator and compared at an equivalent sensitivity. Correlations among the levels were estimated using Pearson’s rank correlation. Statistical analyses were performed using the Statistical Package for Social Sciences 10.0 (SPSS Inc., Chicago, USA). In all tests, a threshold of *P*<0.05 was set for statistical significance.

## Results

### Clinical characteristics

The clinical characteristics of the study groups are shown in Table 1. At blood sampling, maternal age was significantly higher in the SA with fetal chromosomal aneuploidy than in the controls (*P*<0.001). However, there were no differences in the gestational age, body mass index (BMI), and gravidity at blood sampling (*P*>0.05 for all). Alcohol intake and tobacco use were also not different among the three groups (*P*>0.05 for all). Out of a total of 67 SA patients, 41 (61.2%) had fetal normal karyotypes, and 26 (38.8%) showed fetal chromosomal aneuploidy (Table 2). All cases except blighted ovum showed positive FHR prior to maternal blood sampling. The average time difference between maternal blood sampling and the diagnosis of SA was 7 ± 3 days.

**Table 1:** Clinical characteristics of the study population.

Characteristics	SA with fetal chromosomal aneuploidy (n = 26)	SA with fetal normal karyotype (n = 41)	Control (n = 207)	*P-*Value
Maternal age (years)	34.2 ± 4.1^a^	33.1 ± 3.5	32.0 ± 3.6	0.004
Gestational age (weeks)	7.4 ± 1.5	7.3± 1.6	7.9 ± 2.1	0.126
Body mass index (kg/m^2^)	21.9 ± 4.3	22.0 ± 3.9	21.4 ± 2.9	0.499
Gravidity	2.3±1.4	2.1±1.6	2.2±1.2	0.651
Alcohol intake	5 (19.2)	7 (17.1)	32 (15.5)	0.869
Tobacco use	4 (15.4)	6 (14.6)	25 (12.1)	0.828

SA, spontaneous abortion.

Data are presented as mean ± S.D. or number (%).

a SA with fetal chromosomal aneuploidy versus controls, *P*  =  0.033

**Table 2:** Spontaneous Abortion Cases with Fetal Chromosomal Aneuploidy.

Karyotype	Number of patients
45,X	3
46,X,+13	1
46,XY,der(22;22),+22	1
47,XX,+2	2
47,XY,+2	2
47,XX,+8	1
47,XX,+13	1
47,XY,+13	1
47,XX,+14	1
47,XX,+16	3
47,XY,+16	1
47,XX,+21	3
47,XX,+22	4
48,XX,+16,+20	1
69,XXY	1
Total	26

### Levels of cell-free fetal DNA and cell-free total DNA

**Figure 1:**
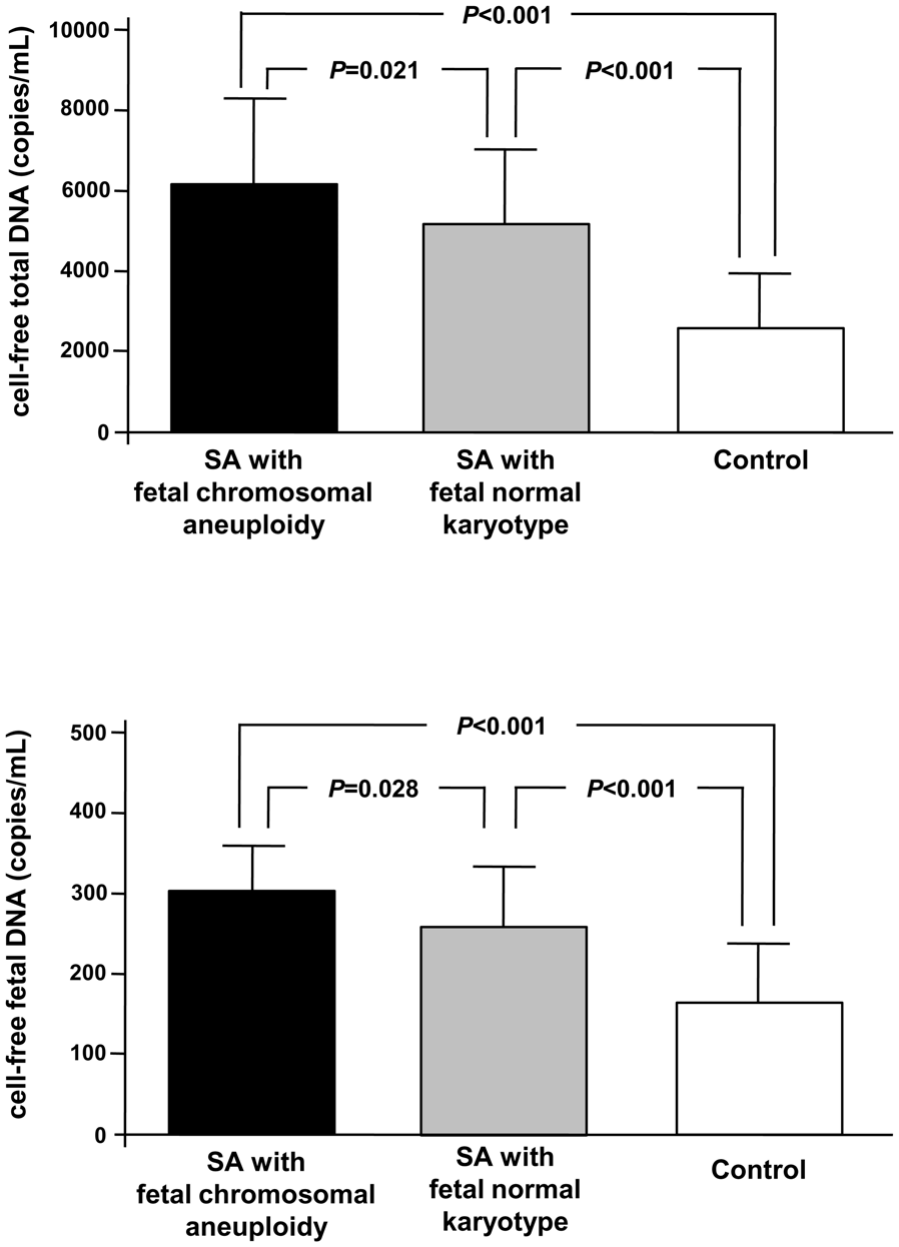
Comparisons of cell-free fetal DNA and cell-free total DNA levels among spontaneous abortion subgroups and controls. Spontaneous abortion (SA) is divided into SA with fetal chromosomal aneuploidy and SA with fetal normal karyotype based on fetal karyotype. The bars and lines upper bars indicate the means and standard deviation, respectively. Data were compared by robust one-way analysis of variance (ANOVA), the post hoc Tamhane’s T2 test for multiple comparisons. A) cell-free total DNA. B) cell-free fetal DNA.

We measured levels of cell-free fetal DNA and cell-free total DNA without PCR failure in all cases. Levels of cell-free fetal DNA and cell-free total DNA are shown in Fig. 1. Levels of cell-free fetal DNA and cell-free total DNA were significantly higher in SA with fetal normal karyotype than in control (253.8 vs. 160.6 copies/mL, respectively, for cell-free fetal DNA, and 5046.4 vs. 2608.4 copies/mL for cell-free total DNA; *P*<0.001 for both). SA participants with fetal chromosomal aneuploidy also showed higher levels of cell-free fetal DNA and cell-free total DNA when compared with control participants (306.9 vs. 160.6 copies/mL, respectively, for cell-free fetal DNA, 6428.3 vs. 2608.4 copies/mL for cell-free total DNA; *P*<0.001 for both). In the analysis of SA according to fetal karyotype, levels of cell-free fetal DNA and cell-free total DNA were significantly higher in SA with fetal chromosomal aneuploidy than in SA with normal karyotype (*P*<0.05 for both, Fig. 1).

### Correlation between cell-free fetal DNA and cell-free total DNA

**Figure 2:**
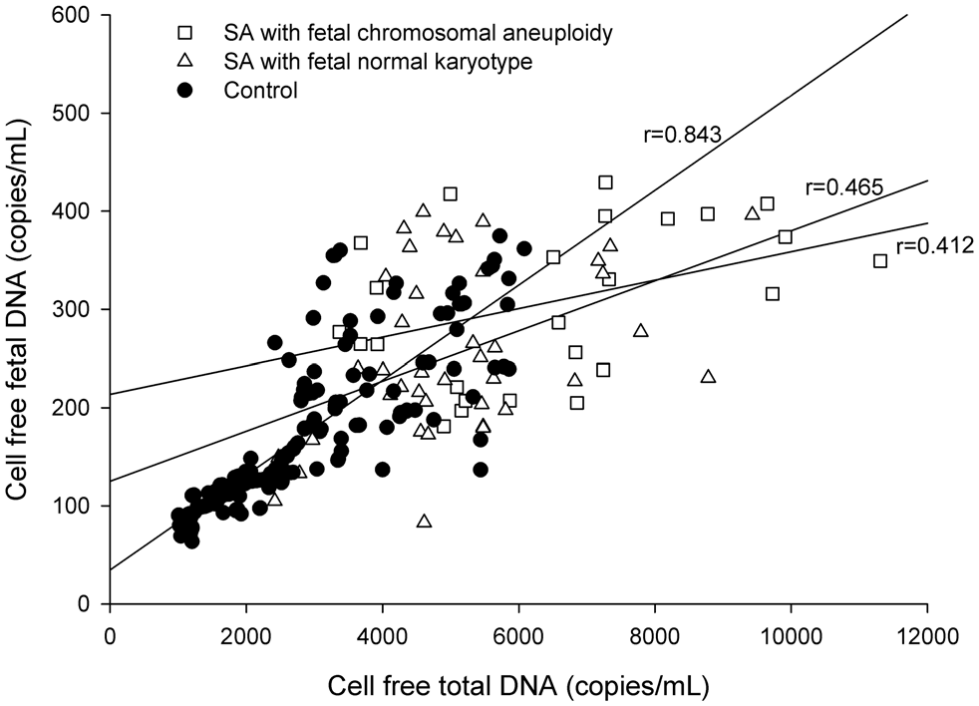
Correlation between cell-free fetal DNA and cell-free total DNA levels in spontaneous abortion subgroups and controls. Cell-free fetal DNA and cell-free total DNA levels showed significantly positive associations in all groups (*P*<0.05). In the analysis among each group, the correlation coefficient between cell-free fetal DNA and cell-free total DNA showed the highest value in the control group. Correlations among the concentrations were estimated using Pearson’s rank correlation.

The level of cell-free fetal DNA was positively correlated with the level of cell-free total DNA in total subjects (r = 0.803, *P*<0.001). In the analysis among each group, the correlation coefficient between cell-free fetal DNA and cell-free total DNA showed the highest value in control (control: r = 0.843, *P*<0.001; SA with fetal normal karyotype: r = 0.465, *P* = 0.002; SA with fetal chromosomal aneuploidy: r = 0.412, *P* = 0.037, Fig. 2). However, levels of cell-free fetal DNA and cell-free total DNA was not correlated with gestational weeks at blood sampling (cell-free fetal DNA: r = 0.003, *P* = 0.972; cell-free total DNA: r = 0.042, *P* = 0.567).

### Prediction of SA women with fetal chromosomal aneuploidy

**Figure 3:**
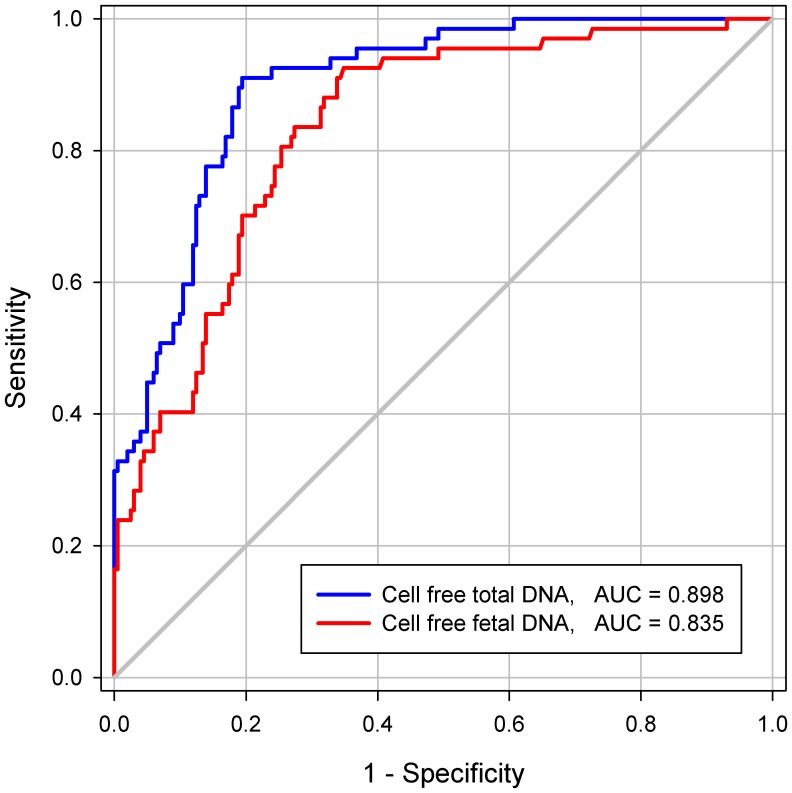
The ROC curves of cell-free fetal DNA and cell-free total DNA levels in total spontaneous abortion. The ROC curves of cell-free fetal DNA and cell-free total DNA levels are represented by red and blue lines, respectively.

**Figure 4:**
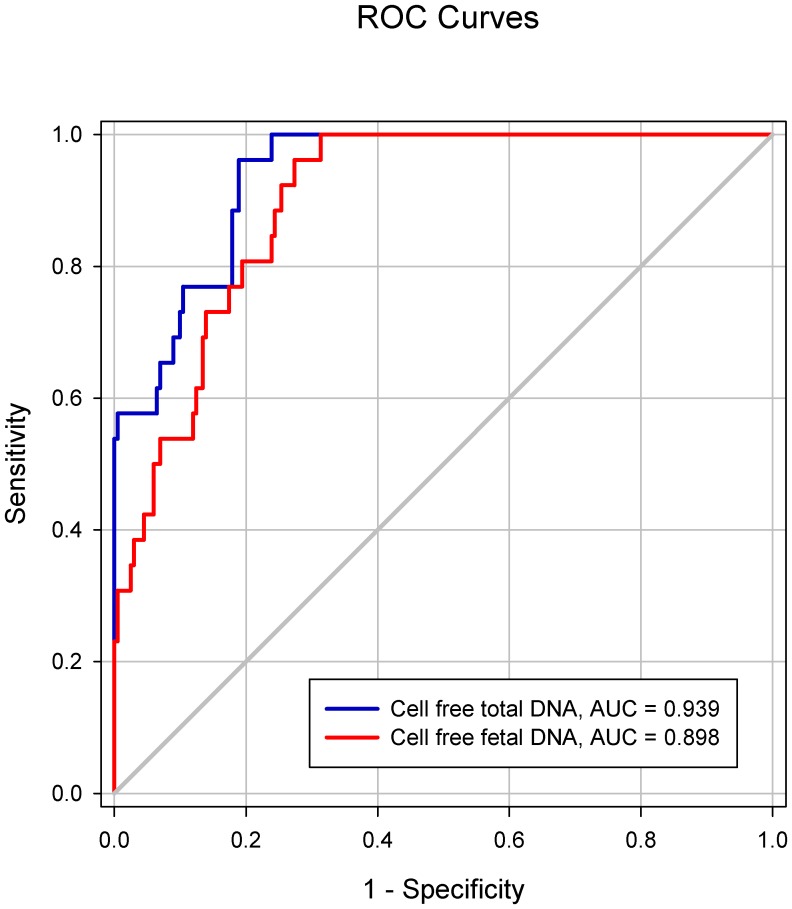
The ROC curves of cell-free fetal DNA and cell-free total DNA levels in spontaneous abortion with fetal chromosomal aneuploidy. The ROC curves of cell-free fetal DNA and cell-free total DNA levels are represented by red and blue lines, respectively.

The accuracies of cell-free fetal DNA and cell-free total DNA in predicting SA during the first trimester are shown in Table 3. The cutoff value for each factor was set at 80% sensitivity by ROC analysis. Specificity, PPV, and NPV were compared at an equivalent sensitivity. The ROC curves of cell-free fetal DNA and cell-free total DNA in SA during the first trimester are presented in Fig. 3. The cutoff level of cell-free fetal DNA was 203 copies/mL, with a specificity of 74.6%, PPV of 51.4%, and NPV of 92.0%. The AUC of cell-free fetal DNA was 0.835 (95% CI: 0.782–0.888) with a standard error (SE) of 0.027 (*P*<0.001). The cell-free total DNA cutoff value of 4,026 copies/mL had a specificity of 83.1%, PPV of 61.4%, NPV of 92.8%, and the AUC was 0.898 (95% CI: 0.859–0.937) with an SE of 0.020 (*P*<0.001). We also analyzed the accuracy of cell-free fetal DNA and cell-free total DNA for predicting SA with fetal chromosomal aneuploidy. The ROC curves of cell-free fetal DNA and cell-free total DNA in SA with fetal chromosomal aneuploidy are presented in Fig. 4. The AUCs of cell-free fetal DNA and cell-free total DNA were 0.898 (95% CI: 0.852–0.945) with a SE of 0.024 and 0.939 (95% CI: 0.903–0.975) with a SE of 0.018 (*P*<0.001 for both), respectively.

**Table 3:** The utility of measurements for detection of spontaneous abortion.

	Cutoff	Specificity (95% CI)	PPV (95% CI)	NPV (95% CI)
Cell-free fetal DNA (copies/mL)	203	0.746 (0.712–0.772)	0.514 (0.449–0.564)	0.920 (0.878–0.952)
Cell-free total DNA (copies/mL)	4,026	0.831 (0.798–0.856)	0.614 (0.539–0.671)	0.928 (0.891–0.956)

CI, confidence interval

The cutoff was set at 80% sensitivity by ROC curve analysis. To compare detection accuracies of factors, sensitivity, positive predictive value (PPV), and negative predictive value (NPV) were calculated at equivalent false positive rate.

## Discussion

Our findings demonstrate that cell-free fetal DNA and cell-free total DNA are effective biomarkers for the noninvasive prediction of SA during the first trimester of pregnancy. We found that cell-free fetal DNA and cell-free total DNA levels were significantly elevated in pregnant women with SA compared to normal pregnant women in the first trimester, regardless of fetal gender. Increased levels of cell-free fetal DNA and cell-free total DNA in maternal circulation showed a high accuracy for prediction of SA. Moreover, detection rates were increased in SA with fetal chromosomal aneuploidy. Therefore, we suggest that cell-free fetal DNA and cell-free total DNA may be useful biomarkers for the prediction of SA with fetal chromosomal aneuploidy during the first trimester, regardless of fetal gender.

The current test to diagnose SA is ultrasound scanning using sonographic parameters such as FHR and CRL. For FHR, low FHR (≤ 120 bpm) is associated with a high likelihood of pregnancy loss and has a high diagnostic accuracy (54.2% sensitivity and 94.8% specificity) [3]. For CRL, a deficit in gestation is associated with an increased incidence of SA [4], [5]. However, at least one repeated ultrasound scanning is usually necessary for effective diagnostic and prognostic detection of SA. Up to 20% of pregnancies miscarry after the visualization of FHR by ultrasonography [7]. Moreover, since the CRL is measured in embryos only, a mistake of merely a few millimeters results in a large deviation.

In a prior study, Yin et al. reported that cell-free fetal DNA and cell-free total DNA levels in SA were significantly higher than those of normal controls by about 5- and 4-fold, respectively [16]. Using cell-free total DNA to predict SA, highly accurate rates of 98.2% sensitivity with a 4.7% false positive rate were detected. On the other hand, using cell-free fetal DNA to predict SA, the sensitivity was 97%, but the false positive rate was as high as 44.3%, as there was a good deal of overlap in the cell-free fetal DNA levels between SA and normal controls. They used the *DYS14* assay to measure the cell-free fetal DNA levels in maternal plasma in a prior study [16]. The *DYS14* assay targets a multicopy sequence on the Y chromosome. However, it can induce nonspecific amplification of artifacts, such as primer dimerization and instability of the fluorescence probe, at a high Cq value [22], [29]. Therefore, this characteristic of the *DYS14* marker might have a considerable effect on the high-false positive rate in prediction of SA using cell-free fetal DNA. Moreover, prior studies are limited to prediction of SA in male fetuses because markers specific to the Y chromosome, such as *DYS14*and *SRY*, were used in the measurement of cell-free fetal DNA [15], [16].

Recently, epigenetic modifications such as fetal-specific signatures have been investigated to overcome limitation of cell-free fetal DNA according to the fetal gender. The human placenta carries a specific DNA methylation pattern that is different with somatic tissues [30], [31]. The majority of cell-free fetal DNA in the maternal plasma is derived from the placental cells, while the cell-free maternal DNA in the maternal plasma is derived from the maternal hematopoietic cells [17]–[19]. Therefore, genomic regions that are differentially methylated between the placenta and the maternal blood cells have been considered as fetal-specific epigenetic makers in maternal plasma. To detect fetal epigenetic markers in maternal plasma, the first step is to differentiate methylated and unmethylated sequences. Various methods, such as a bisulfite modification of the template DNA, differential cleavage by restriction enzymes and antibody-mediated enrichment of methylated fragments by methylated DNA immunoprecipitation, are applied. The next step is to quantify a fetal-specific methylation marker. In general, PCR-based methods, such as qMSP and quantitative real-time PCR, are used. Through this process, cell-free fetal DNA can be measured selectively in maternal blood. In this study, we used the *U-PDE9A* for the measurement of the level of circulating cell-free fetal DNA in maternal plasma. Because the region of *PDE9A* is completely methylated in the maternal blood and unmethylated in fetal (placental) tissues [20]–[22]. Therefore, the unmethylated CpG sequences in *PDE9A* target region were modified by bisulfite conversion and selectively amplified by qMSP. As a result, only cell-free fetal DNA levels could measure in all pregnant women regardless of fetal gender.

In our previous study, we reported that cell-free fetal DNA and cell-free total DNA showed a strong positive correlation in normal pregnant women [22]. Other investigators also reported a positive correlation between cell-free fetal DNA and cell-free total DNA to a greater or lesser extent depending on the method and markers used [32]. These findings demonstrated that the levels of cell-free fetal and cell-free total DNA have a proportional characteristic in maternal blood in normal pregnant women. However, in this study, we found that the correlation coefficient between cell-free fetal DNA and cell-free total DNA was lower in SA women than in normal pregnant women. This may result from an abnormal increase in cell-free fetal DNA and cell-free total DNA when SA occurs. Additionally, we analyzed the correlation between level of cell free fetal DNA and cell free total DNA and gestational weeks. Level of cell free fetal DNA and cell free total DNA were not correlated with gestational weeks, a finding that consistent with previous study [32]. This might be explained by fact that sampling duration (5–12 weeks of gestation) of maternal blood was not a sufficient period to determine association between cell-free DNAs and gestational weeks.

Prior studies have reported that an increase in cell-free total DNA, as well as cell-free fetal DNA, is associated with SA [15], [16]. This increase in cell-free total DNA may be induced by the release of circulating factors into maternal circulation, which can result in damage to maternal vascular endothelial cells and multi-system dysfunction [10]. Other explanations include the increase in size of the fetomaternal interface as gestation progresses and a reduction in DNA clearance associated with other physiologic changes during pregnancy [27]. When SA occurs, increased levels of maternal plasma cell-free fetal DNA can result in a breakdown of the placental barrier and an abnormal separation of the placenta [10]. In the present study, we also found that cell-free fetal DNA and cell-free total DNA levels were significantly increased in SA cases compared to controls. These results were consistent with those from previous studies [15], [16]. Additionally, we demonstrated that the use of cell-free fetal DNA and cell-free total DNA was more informative in predicting SA with fetal chromosomal aneuploidy than SA with fetal normal karyotype. Using cell-free fetal DNA and cell-free total DNA to predict SA, the AUCs were 0.835 and 0.898, respectively. On the other hand, using cell-free fetal DNA and cell-free total DNA to predict SA with fetal chromosomal aneuploidy, the AUCs were 0.898 and 0.939, respectively. These may be a result of the reduction in the overlap range due to higher levels of cell-free fetal DNA and cell-free total DNA in SA with fetal chromosomal aneuploidy than in SA with fetal normal karyotype.

In prior studies, cell-free fetal DNA could predict the outcome of women carrying a male fetus only, as the *DYS14* and *SRY* sequences are Y-specific sequences [15], [16]. In the present study, we used a fetal-specific epigenetic marker such as *U-PDE9A* to measure cell-free fetal DNA levels in maternal plasma. Therefore, the applicable range of cell-free fetal DNA for prediction of SA was extended to all pregnant women, regardless of fetal gender. However, this epigenetic marker might have potential errors such as CpG dinucleotide content in target regions and methylation difference in developmental process of placenta that could influence the level of methylation in the cell-free fetal DNA. Therefore, it is important to confirm whether methylation pattern of this marker is changed according to developmental process of placenta and CpG dinucleotide content is optimal in PCR performance. In the *PDE9A* used in this study, its tissue-specific methylation pattern was confirmed in maternal bloods and placentas obtained from both the first and third trimesters [20] and the target region of *U-PDE9A* for PCR showed a high PCR efficiency [22].

In conclusion, we have found that both maternal plasma cell-free fetal DNA and cell-free total DNA levels increased in SA women with fetal chromosomal aneuploidy. We demonstrated that the use of cell-free fetal DNA and cell-free total DNA was informative in noninvasive predicting of SA with fetal chromosomal aneuploidy. Therefore, we suggest that cell-free fetal DNA and cell-free total DNA may be useful biomarkers for the prediction of SA with fetal chromosomal aneuploidy during the first trimester of pregnancy. Although it is unlikely that cell-free fetal and total DNA measurements will replace ultrasound in the near future, improved prediction will provide opportunities for potential intervention. Therefore, we suggest that this technology may be useful in clinical settings for the prediction and diagnosis of SA with fetal chromosomal aneuploidy. Eventually, we believe that the availability of a reliable noninvasive test to determine SA with fetal chromosomal aneuploidy would reduce unintended fetal loss and would presumably be welcomed by pregnant women at risk for SA. Moreover, our results are helpful in understanding the molecular mechanisms of cell-free fetal DNA and cell-free total DNA in early pregnancy loss with fetal chromosomal aneuploidy. However, this method needs to be further refined to achieve higher sensitivity and specificity by combination with other biomarkers such as cytokines, etc. Furthermore, this study is limited by its small sample size and inclusion of only Korean patients. Therefore, our results need to be confirmed by further studies on a larger-scale cohort of patients within different ethnic populations.
